# Supplementary material for: Wnt Modulation Enhances Otic Differentiation by Facilitating the Enucleation Process but Develops Unnecessary Cardiac Structures
Source: Int J Mol Sci. 2021 Sep 24;22(19):10306. doi: 10.3390/ijms221910306 (PMC8508801; doi:10.3390/ijms221910306)
Supplement: Supplementary file 1 [file ijms-22-10306-s001.zip › Supplementary Table S1 ijms.pdf]

Supplementary Table S1. Statistical analysis of levels of mRNA expression for HC differentiation-related genes using RT-qPCR.

Samples	(P Value)	Target Genes						
		SOX2	E-Cad	Laminin	PAX2	PAX8	ATOH1	M7A
CHIR vs. (−) Control	P > 0.05 (ns) P < 0.001 (***)	***	ns	***	***	***	***	***
CHIR vs. (+) Control 7D	P > 0.05 (ns) P < 0.001 (***)	***	ns	***	***	***	***	***
CHIR vs. (+) Control 14D	P > 0.05 (ns) P < 0.001 (***)	ns	ns	ns	ns	ns	ns	***
